# Metformin-mediated intestinal AMPK activation ameliorates PCOS through gut microbiota modulation and metabolic pathways

**DOI:** 10.3389/fendo.2025.1526109

**Published:** 2025-02-18

**Authors:** Yating Xu, Li Ning, Yu Si, Xiu Li, Ruyue Wang, Qingling Ren

**Affiliations:** The Affiliated Hospital of Nanjing University of Chinese Medicine, Nanjing, China

**Keywords:** polycystic ovary syndrome, AMP-activated protein kinase, Indole-3-carboxaldehyde, gut microbiota, serum metabolites

## Abstract

**Introduction:**

Polycystic ovary syndrome (PCOS) is a complex disorder characterized by metabolic and ovulatory dysfunctions, often associated with an imbalance in gut microbiota. Despite current treatments, effective management strategies targeting underlying mechanisms remain limited.

**Methods:**

In this study, we used a rat model of PCOS induced by letrozole and a high-fat diet. The effect of intestinal AMP-activated protein kinase (AMPK) activation was evaluated through metformin administration, the most commonly used AMPK activator. We analyzed metabolic parameters, ovulatory functions, gut microbiota composition, and serum levels of Indole-3-carboxaldehyde (I3A), a metabolite involved in inflammation and apoptosis regulation.

**Results:**

Metformin treatment significantly reversed metabolic disorders and restored ovulatory functions in PCOS rats. Moreover, metformin treatment led to notable improvements in gut microbiota composition and an increase in serum I3A levels, which have been shown to mitigate inflammation and apoptosis.

**Discussion:**

This study highlights the therapeutic potential of targeting intestinal AMPK in managing PCOS. By improving both metabolic and reproductive health, activation of AMPK may offer a promising approach for restoring physiological balance in PCOS patients.

## 1 Introduction

Polycystic Ovary Syndrome (PCOS) affects 10%-13% of women of reproductive age and is a prevalent endocrine disorder (1). PCOS is characterized by ovarian dysfunction, polycystic ovarian morphology, and hyperandrogenemia. Dysfunctional ovulation in PCOS frequently results from restricted proliferation of ovarian granulosa cells, leading to excessive apoptosis in the follicular granulosa cell layer, follicular arrest, and subsequent polycystic ovarian changes (2). This mechanism is considered critical in the pathogenesis of PCOS. Furthermore, significant disturbances in gut microbiota are observed in patients with PCOS (3). However, the underlying mechanisms and the interaction between gut microbial disturbance and ovulatory dysfunction in PCOS warrant further investigation.

AMP-activated protein kinase (AMPK), a crucial enzyme in eukaryotic cells, is activated by phosphorylation through upstream kinases and plays a vital role in maintaining cellular energy metabolism stability. AMPK is widely existed across mammalian tissues, inhibiting cholesterol synthesis in the liver (4) and inducing fat browning in adipose tissue (5). Recent research has increasingly focused on the role of intestinal AMPK in metabolic syndrome and related diseases (6). Intestinal AMPKα1-IKO mice has significantly altered gut microbiota (7). Metformin is the most commonly used activator of AMPK (8). It is also widely used in clinical practice to treat polycystic ovary syndrome to improve insulin resistance, maintain glucose homeostasis, and reduce the risk of metabolic complications (9–11). Given the potentially significant role of intestinal AMPK activation in PCOS treatment with metformin, this study aims to further explore the mechanisms of intestinal AMPK activation in metabolic regulation and its impact on treatment strategies.

## 2 Results

### 2.1 AMPK activation reverses metabolic disorders and ovulatory dysfunction in rats with PCOS

**Figure 1:**
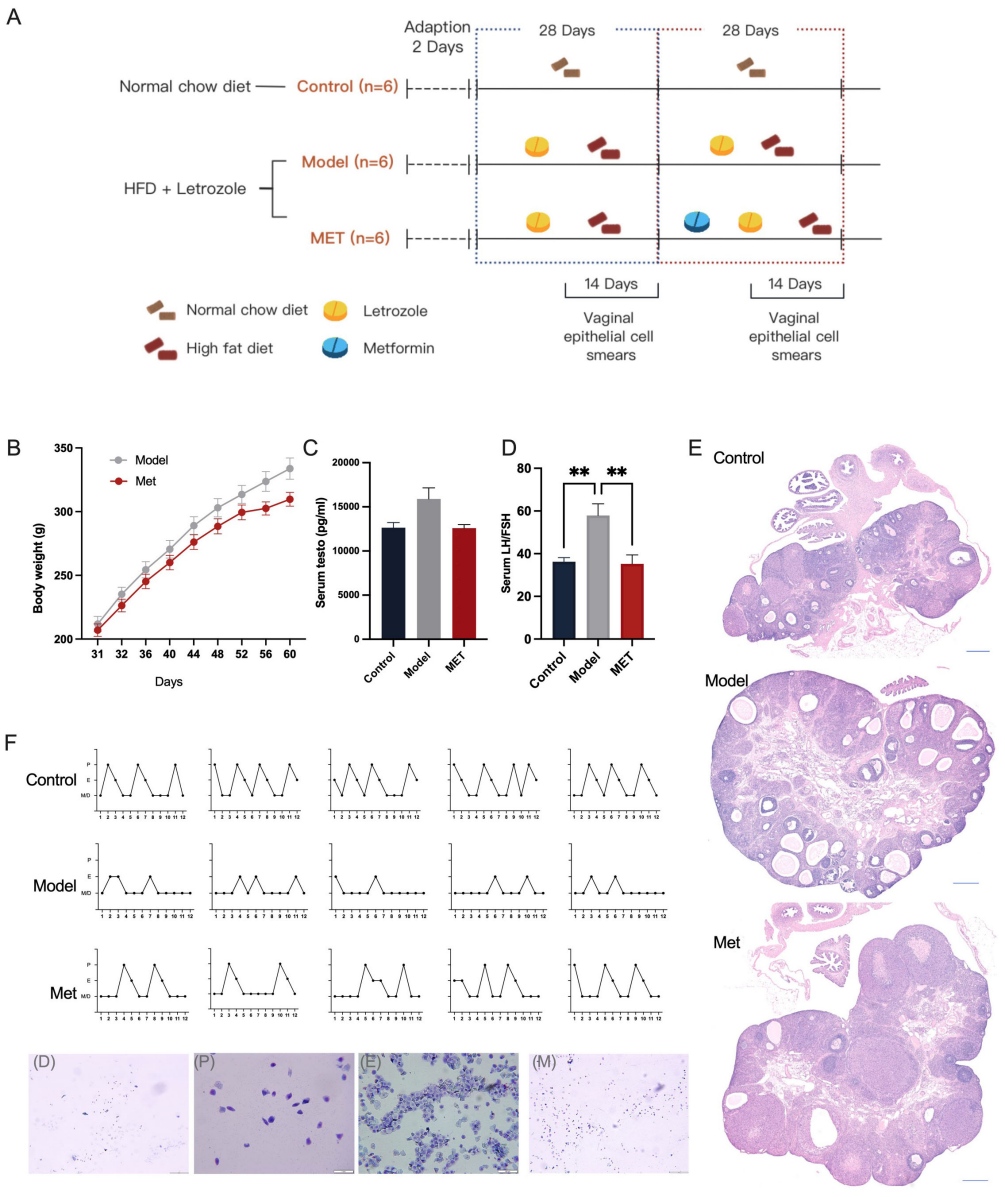
AMPK activation reverses metabolic disorders and ovulatory dysfunction in rats with PCOS. The experimental grouping and timeline are as depicted in Figure **(A)**. Female rats were treated with letrozole combined with a high-fat diet for 21 days, followed by gavage with Metformin or H2O for another 21 days. The rats’ weights were monitored daily (n=6) **(B)**. Serum testosterone and LH/FSH levels were measured using radioimmunoassay and enzyme-linked immunosorbent assay kits respectively **(C, D)** (n=6). The estrous cycle was determined 14 days post-treatment (n=5) **(F)**. Representative ovarian sections were stained with hematoxylin and eosin (scale bar = 500μm) **(E)**. Data are presented as mean ± standard error of the mean (SEM); * indicates significance at p<0.05; ** indicates significance at p<0.01.

We successfully established a rat model of polycystic ovary syndrome using letrozole combined with a high-fat diet, which is a PCOS model exhibiting metabolic disorders (12) (**Figure 1A**). The study included three groups: Control (Control, n=6), Model (Model, n=6), and Metformin intervention (Met, n=6). For the first 28 days, both the Model and Metformin intervention groups were treated with letrozole and supplemented with high-fat diet. Subsequently, for the next 28 days, the Metformin intervention group received metformin via gavage.

By the study’s end, rats in the Model group exhibited significant alterations in the LH/FSH ratio (mean ± SEM: Control: 36.26 ± 1.922, Model: 57.91 ± 5.425, Met: 35.22 ± 2.206), elevated testosterone levels (mean ± SEM: Control: 12635 ± 583.6, Model: 15901 ± 1246, Met: 12591 ± 399.9), and weight gain (**Figures 1B-D**). H&E staining of the ovaries in the Model group revealed polycystoid changes (**Figure 1E**), an increased number of follicles, and disturbances in the estrous cycle (**Figure 1F**), indicating successful modeling.

Previous studies have demonstrated that mice with targeted intestinal AMPK knockout exhibit significant metabolic disorders (7). To evaluate the role of AMPK in PCOS, we used metformin, an AMPK activator, to address metabolic disorders in rats with PCOS. Following metformin treatment, metabolic disorders and ovarian dysfunction were ameliorated in the PCOS model group.

### 2.2 Metformin activates intestinal AMPK, alleviating the impact of PCOS on gut microbiota and antimicrobial peptides

**Figure 2:**
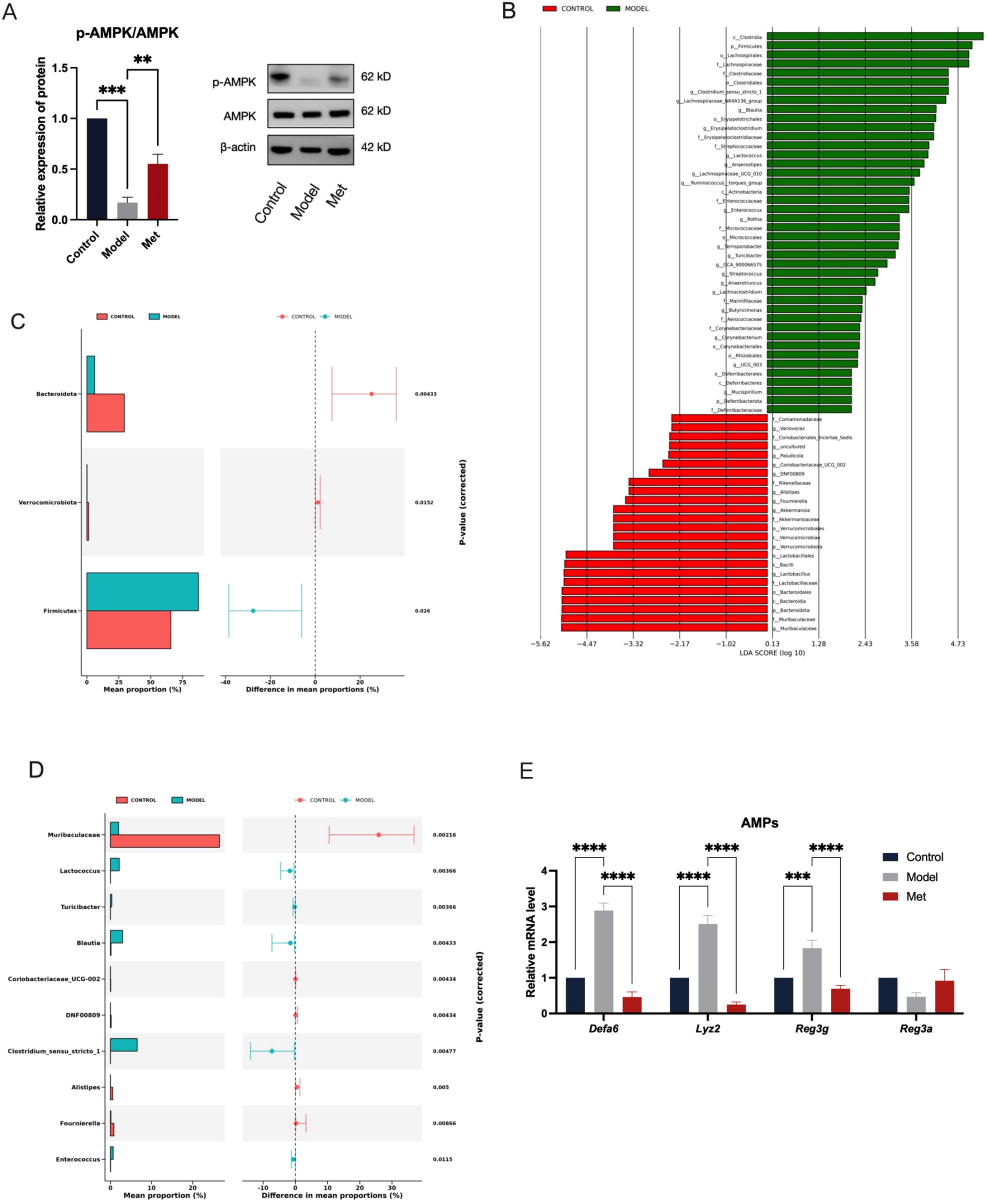
Metformin Activates Intestinal AMPK, Alleviating the Impact of PCOS on Gut Microbiota and Antimicrobial Peptides. The ileal tissue lysates were examined using Western blotting to determine the expression levels of p-AMPK and AMPK, with β-actin serving as the loading control **(A)** (n=6). The Linear Discriminant Analysis (LDA) Effect Size (LEfSe) method was used to identify statistically significant differences in biomarker levels between the Control (red) and Model (green) groups **(B)**. Only functional entries with an LDA Score greater than a set threshold of 2 are displayed; the length of the bars represents the magnitude of the LDA values **(C, D)**. These graphs show the abundance ratios of different species in the two samples or sample groups, with the middle indicating the difference ratio within the 95% confidence interval, and the value on the far right is the p-value, with p < 0.05 indicating a significant difference **(E)**. Relative mRNA levels of antimicrobial peptides in the ileum of rats in the Control, Model, and Met groups (n = 6). * indicates significance at p<0.05; ** at p<0.01; *** at p<0.001;**** at p<0.0001.

Upon measuring intestinal AMPK protein levels, we observed significant suppression in PCOS model rats, notably in the ratio of phosphorylated to total AMPK (p-AMPK/AMPK) as shown in **Figure 2A** (mean ± SEM: Model: 0.1692 ± 0.05284, Met: 0.5515 ± 0.09490). Following oral metformin administration, AMPK activity in rat intestines was activated, leading to an upregulation of protein expression. Restoration of intestinal AMPK in PCOS rats led to marked improvements in various disease indicators (**Figures 1B-F**). These findings suggest that metformin alleviates the disease phenotype in PCOS rats by activating intestinal AMPK, highlighting its potential as a target for PCOS treatment.

We examined gut bacteria abundance in rats with PCOS and found that the PCOS model disrupts intestinal microbiota (**Figures 2B-E**). Using a significance threshold of 0.05, we identified significant differences in bacterial phyla: Bacteroidota, Verrucomicrobiota, and Firmicutes (**Figure 2C**). At the genus level, significant differences were observed in several bacteria: Muribaculaceae, Lactococcus, Turicibacter, Blautia, Coriobacteriaceae_UCG-002, DNFO0809, Clostridium_sensu_stricto_1, Alistipes, Fournierella, Enterococcus (**Figure 2D**). At the phylum level, the distribution of Firmicutes, Bacteroidota, and Verrucomicrobiota indicates the overall composition of gut microbiota in an organism, with Firmicutes predominately comprising harmful bacteria, and Bacteroidota enriched with beneficial bacteria. Although Verrucomicrobiota comprises only a small fraction of the total gut microbial community, it plays a critical role in metabolic and inflammatory diseases. Compared with the Control group, there was a significant increase in the abundance of Firmicutes in the Model group, while the abundances of Bacteroidota and Verrucomicrobiota were significantly reduced (**Figure 2C**). LEfse analysis revealed that higher LDA values correlate with more significant differences between groups. Bacteroidota, Muribaculaceae, and Clostridia were identified as the genera with the most significant differences between the Control and Model groups (**Figure 2B**).

Previous studies have linked metformin treatment of PCOS with changes in gut microbiota, suggesting that AMPK activation may influence goblet cell secretion of antimicrobial peptides, thereby affecting gut microbiota homeostasis. We measured mRNA levels of gut antimicrobial peptides. Accompanying a decrease in AMPK expression, the PCOS rat ileum exhibited significant up-regulation of antimicrobial peptides, which was reversed by the AMPK-activating agent metformin (**Figure 2E**). It has been shown that while antimicrobial peptide secretion typically improves intestinal mucosal barriers, excessive amounts can contribute to intestinal inflammation (13). Thus, we hypothesized that metformin administration may act by activating intestinal AMPK, modulating the expression of antimicrobial peptides, and balancing the homeostasis of the gut microbiota in PCOS.

### 2.3 Gut microbiota derived metabolites: Indole-3-Carboxaldehyde

**Figure 3:**
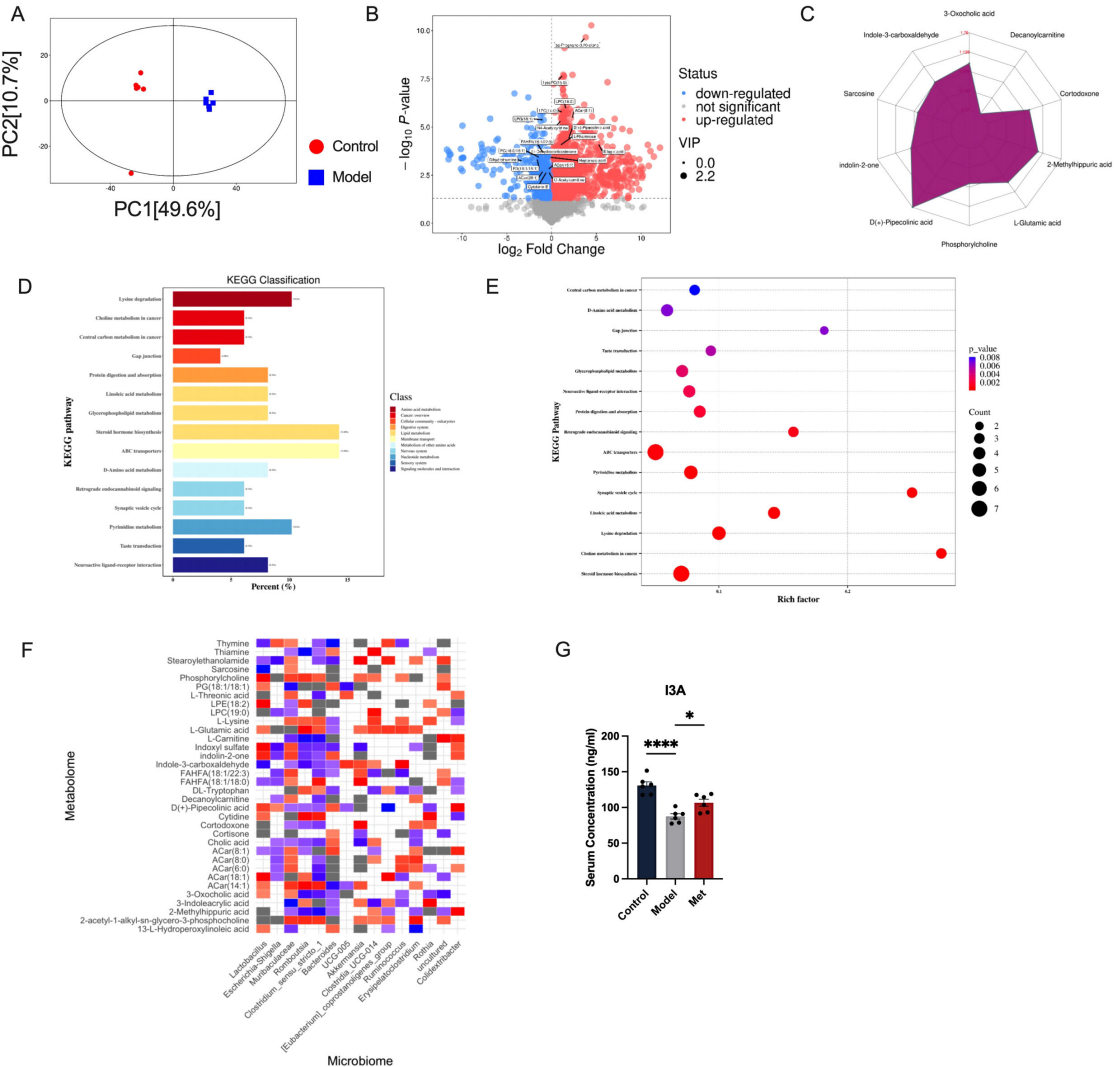
Correlation analysis between intestinal bacteria and serum metabolites **(A)** Score scatter plot of PCA model for group Control vs Model. The x-axis labeled PC (1) and the y-axis labeled PC (2) represent the scores of the first and second principal components respectively. Each dot in the plot represents a sample, with the Control group in red and the Model group in blue. The closer the distribution of sample points, the more similar are the types and contents of metabolites within the samples; conversely, the greater the distance between sample points, the greater the overall metabolic level differences **(B)**. Each point in the plot represents a peak, including all substances measured in this experiment. The x-axis represents the fold change of each substance in the group comparison (logarithm base 2), the y-axis represents the p-value of the Student’s t-test (negative logarithm base 10), and the size of the dots represents the VIP values from the OPLS-DA model; larger dots indicate higher VIP values. Metabolites that are significantly upregulated are shown in red, significantly downregulated metabolites in blue, and metabolites with no significant differences in gray **(C)**. Quantitative values of differential metabolites are calculated for their ratios and converted using the logarithm base 2, shown in red font in the graph. Each grid line represents a difference multiplier, with the purple shadow composed of lines connecting the difference multipliers of each substance, displayed in a radar chart to show the trend of content changes **(D)**. The x-axis represents the percentage of annotated differential metabolites in a pathway relative to all annotated differential metabolites, while the y-axis lists the names of enriched KEGG metabolic pathways **(E)**. The x-axis represents the Rich Factor of each pathway, and the y-axis names the KEGG metabolic pathways. The size of the dots indicates the number of differential metabolites enriched in the pathway. The color indicates the p-value; smaller p-values are shown in redder colors, indicating a more significant enrichment **(F)**. Red squares indicate strong correlations between the microbial community and metabolites in the Model group. Blue squares indicate strong correlations in the Control group. Grey squares or colorless sections indicate no significant differences in correlations between the microbial communities and metabolites between the Control and Model groups (n=6). **(G)** shows that the Control group has the highest serum concentration of I3A, followed by the Model group with a lower concentration, and the Met group with a concentration slightly higher than the Model group (n=6, mean ± SEM: Control: 130.8 ± 5.145, Model: 87.51 ± 3.715, Met: 106.5 ± 4.993). Statistical significance is indicated by the asterisks, * indicates significance at p<0.05; **** at p<0.0001.

We further explored how changes in gut microbiota might affect the ovaries of PCOS rats. Gut microbial serum metabolites serve as intermediates in host-gut microbiota interactions. Serum metabolomics conducted on both blank and PCOS model rats revealed significant differences in serum metabolites in the PCOS group (**Figure 3A**). In PCOS rats, levels of LPC(16:2), LPC(14:0), ACar(8:1), N4-Acetylcytidine, D(+)-Pipecolinic acid, and Indole-3-carboxaldehyde were significantly down-regulated (**Figures 3B, C**). KEGG analysis indicated that the changes in serum metabolites were closely linked to steroid hormone biosynthesis, ABC transporters, and other metabolic pathways (**Figures 3D, E**).

In order to explore the correlation between changes in intestinal homeostasis and serum metabolites, we conducted a correlation analysis between gut microbiota and serum metabolites to identify potential targets. Our correlation analysis of gut microbiota-serum metabolites in control and model groups (**Figures 3D-F**) highlighted the tryptophan-related metabolite Indole-3-carboxaldehyde (I3A). Gut microbes play a significant role in tryptophan digestion, and the indole pathway is impaired by a high-fat diet that reduces probiotic abundance (14). In HUVEC cells experiencing oxidative stress, I3A reduces the release of pro-inflammatory cytokines and lowers ROS levels (15). Research has shown that I3A promotes the growth of Lactobacillus Reuteri and prevents obesity induced by a high-fat diet in mice (16). Our findings align with previous results showing that I3A release in healthy SD rats is closely linked to Lactobacillus bacteria (17). We consider that metformin could improve PCOS by improving the structure of intestinal flora, adjusting the abundance of beneficial intestinal bacteria, upregulating serum I3A concentration (**Figure 3G**).

### 2.4 I3A inhibits KGN cell apoptosis, contributing to PCOS recovery

**Figure 4:**
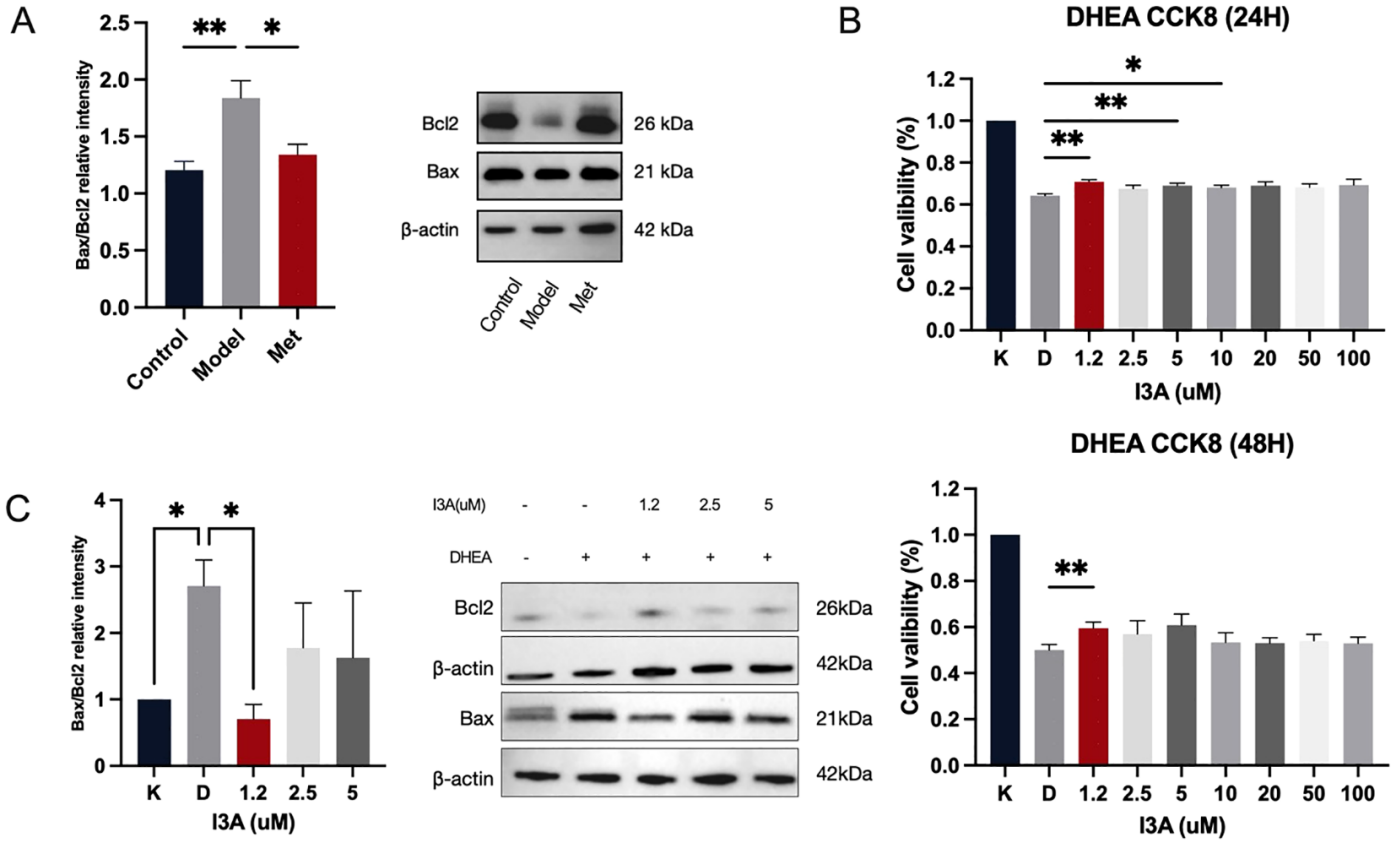
I3A inhibits KGN cell apoptosis, contributing to PCOS recovery. **(A)** Western blotting was used to examine the expression levels of BAX and Bcl2 in the lysates of ovarian tissues from the Control, Model, and Met groups. **(B)** Cell viability (%) of KGN-DHEA cells under different I3A concentration gradients. **(C)** Expression levels of BAX and Bcl2 in cell lysates under different I3A concentration gradients. Data are presented as mean ± standard error of the mean (SEM); * indicates significance at p<0.05; ** indicates significance at p<0.01.

We initially examined the protein expression of the anti-apoptotic gene B-cell lymphoma 2 (BCL2) and the pro-apoptotic gene Recombinant Bcl2 Associated X Protein (Bax) in ovarian tissues of rats in each group. Significant apoptosis was observed in the ovarian tissues of PCOS rats, which metformin effectively mitigated (**Figure 4A**). Next, we investigated whether I3A impacts ovarian granulosa cells and contributes to reversing ovulation disorders in PCOS. I3A is known for its anti-inflammatory, anti-infective, and anti-aging properties (18), and it acts through inhibiting apoptosis; however, its role in ovarian function remains unclear. We used DHEA to intervene with KGN cells *in vitro*. I3A promoted the proliferation of DHEA-treated KGN cells in a dose-dependent manner (**Figure 4B**). At concentrations ranging from 0 to 100 µM, I3A countered the effects of apoptosis and proliferation suppression induced by DHEA in KGN cells (**Figure 4B**). We selected the optimal concentration, along with two adjacent concentrations, to establish a low, medium, and high gradient (1.2 µM, 2.5 µM, 5 µM) for the intervention of KGN cells. We examined the protein expression of Bax and BCL2 at concentrations of 1.2, 2.5, and 5 µM, finding that I3A maximized its anti-apoptotic effect at 2.5 µM, promoting KGN proliferation and protecting ovarian function (**Figure 4C**).

## 3 Discussion

Our study evaluated the effects of metformin on PCOS rats through gut microbiomics and metabolomics, exploring the potential role of intestinal AMPK in gynecological reproductive endocrinology. Our findings indicate that intestinal AMPK plays a crucial role in the onset and treatment of PCOS by regulating gut microbiota homeostasis and enhancing the secretion of the gut microbial metabolic product I3A in serum. Metformin significantly ameliorates ovarian apoptosis in PCOS rats and promotes the level of I3A. *In vitro* experiments further confirmed that I3A can rescue apoptosis induced by DHEA in KGN cells and promote their proliferation to some extent.

AMPK is a vital component of cellular energy metabolism (19), extensively studied for its role in the development of PCOS. Diminished AMPK activity accompanied by impaired fatty acid cycle was observed in subcutaneus adipose tissue of adolescent rats with PCOS (20). Early gestational androgen exposure in obese mice with PCOS leads to metabolic disturbances in female offspring, accompanied by significantly reduced levels of phosphorylated AMPK in the ovary (21). AMPK expressed in the intestinal mucosa, serves not only as another target of metformin beyond the liver (22) but also plays a critical role in regulating energy intake, expenditure, and weight control (7). Given the intestine’s crucial role in energy uptake and absorption, recent studies have focused on the activation of intestinal AMPK (6, 23), which has been shown to reduce hepatic gluconeogenesis in rats (22), thereby stabilizing metabolic functions. Intestinal AMPK knockout mice showed significant weight gain and disturbed gut microbiota (7). Previous studies have highlighted intestinal AMPK’s key role in metabolic diseases, its activation in the context of reproductive endocrinology remains unexplored. In our study, we found that phosphorylation AMPK protein in the intestine of PCOS rats was attenuated, along with gut microbiota disruption and reduced abundance of beneficial bacterial genera. In contrast, the ovulatory dysfunction and metabolic abnormalities displayed by the model rats were restored after administration of an AMPK activator. The abundance of gut microbiota in rats with PCOS differed from that in normal SD rats (24).

Intestinal AMPK influences the structure of the gut microbiome by regulating the secretion of antimicrobial peptides (25). Beyond their direct bactericidal effects, AMPs also modulate the gut microbiota by drawing various immune cells to the site of infection, enhancing the immune response (26). In our study, the mRNA levels of antimicrobial peptides in the intestines of PCOS rats were significantly higher than those in the healthy control group and the metformin-treated group. This elevation may be partly due to the impact of PCOS on intestinal barrier function, which in turn promotes the secretion of AMPs to some extent; another part may stem from the abnormal intestinal microenvironment affecting the normal expression of AMPs. Additionally, other studies have shown that more AMPs are not always better within the intestine. For instance, the expression of AMPs in the ileum becomes dysregulated in the elderly (27), and excessive AMPs can impair intestinal barrier function, exacerbating symptoms of colitis (28). The homeostasis of the intestinal environment requires a precise balance of AMP expression, where either excessive or insufficient expression can disrupt this balance. Future research will further explore the specific mechanisms behind these findings to clarify the role of AMPs in regulating intestinal health.

Crosstalk between gut microbiota and disease may be mediated by serum metabolites derived from gut microbiota. Our study focused on the significantly different metabolite, indole-3-carboxaldehyde, in the serum of healthy rats versus PCOS model rats by coanalysis. Indole-3-carboxaldehyde, an indole derivative, has anti-inflammatory properties and inhibits apoptosis in cells. Our study found that PCOS rats showed significant ovarian granulosa cell apoptosis that was alleviated by metformin treatment. To further explore the effect of I3A on apoptosis, we chose KGN cells for our study. KGN cells derived from human ovarian granulosa cells are widely used in female reproductive endocrine studies. After 20uM DHEA intervention (29), KGN cells showed significant growth inhibition, while 50uM I3A administration was able to inhibit KGN apoptosis and promote cell proliferation under DHEA modeling.

Our study’s limitations include the absence of gene knockout techniques to confirm the critical role of intestinal AMPK protein. Due to this constraint, we look forward to future studies that might employ gene knockout in female mice to conduct histological and reproductive function assessments of the ovaries, thereby demonstrating the crucial role of intestinal AMPK in female reproductive endocrinology. PCOS is a chronic condition requiring long-term management, thus needs more long-term clinical follow-up evidence to clarify the potential role of I3A in clinical settings.

By establishing a rat model of PCOS, we revealed the mechanism by which metformin ameliorates the pathology of PCOS through activation of intestinal AMPK and pointed out the important role of intestinal microbiota and its metabolite I3A in this process. Our study not only demonstrates the potential role of gut flora in regulating endocrine diseases, but also provides a scientific basis for novel therapeutic strategies for PCOS. In addition, the anti-apoptotic and cell proliferative effects of I3A reveal its potential value in maintaining ovarian health, opening up new research directions and therapeutic possibilities for PCOS treatment. This finding provides new insights into the regulation of ovarian function by gut flora through specific metabolites.

## 4 Conclusion

Our research conclusively demonstrates that metformin’s activation of intestinal AMPK plays a pivotal role in ameliorating the symptoms of PCOS in rats. By modulating gut microbiota and enhancing the production of the beneficial metabolite I3A, metformin effectively reduced ovarian granulosa cell apoptosis and promoted cellular proliferation, thereby restoring ovarian function and metabolic balance. These findings suggest a significant link between gut microbiota and ovarian function, mediated through metabolic pathways influenced by intestinal AMPK. The therapeutic potential of metformin, facilitated through gut microbiome modulation, offers a promising avenue for PCOS treatment and potentially other related metabolic and reproductive disorders.

## 5 Methods

### 5.1 Experimental animals

Female Sprague-Dawley 3-week-old rats (license No. scxk (Jing) 2021-0011) were purchased from Charles River Laboratories. Rats were reared in the SPF barrier system. In this study, environmental conditions were maintained at a temperature of 22 ± 2°C and humidity of 55 ± 10%, with a consistent 12-hour light-dark cycle. The protocol was evaluated and received approval from the animal ethics committee at Nanjing University of Traditional Chinese Medicine, under the approval code 202204A054.

Following a three-day adaptation period during which the animals were acclimatized to their surroundings, the rats were randomly divided into Control, Model and Met groups (n=6 per group). Throughout the duration of the experiment, the Control group rats were provided with standard chow and received daily oral administration of 1% sodium carboxymethyl cellulose. Conversely, the rats in the Model and Met groups were supplied with a high-fat diet (sourced from Research Diets, Inc., New Brunswick, NJ, USA) and were orally administered letrozole continuously over a 21-day period. The letrozole was administered at a dose of 1 mg/kg/day, dissolved in 1% sodium carboxymethyl cellulose.

At the end of modeling, the dose was 0.25 g/kg/day in the metformin group. Control group was given CMC and pure water by gavage for 30 d. Model and Metformin groups were given letrozole in combination with a high-fat diet to maintain the model. Each rat weight was recorded every day. The feces of rats were collected on the last day of modeling and drug treatment, microbial analysis was carried out on fecal samples. Finally, all rats were euthanized after fasting for 12 h and anesthetized with 2% sodium pentobarbital (40 mg/kg). Ovary and small intestine tissues were collected and part of them were fixed with paraformaldehyde. The remaining fresh tissues were treated with liquid nitrogen and stored in a -80°C refrigerator for further experiments. Blood was collected from the abdominal aorta, and serum was centrifuged at 3000 rpm and 4°C for 15 min. All subsequent experiments were conducted with n=6 per group.

### 5.2 Assessment of estrous cycle

The stage of the estrous cycle in the subjects was assessed daily through the examination of vaginal epithelial cell smears using a light microscope. Classification of the estrous cycle phase was based on the presence and types of cells observed, specifically leukocytes, cornified epithelial cells, and nucleated epithelial cells.

### 5.3 Elisa

Serum luteinizing hormone (LH, LA166603H), follicle stimulating hormone (FSH, LA166605H), testosterone (T, LA182202H) levels were determined using commercially available ELISA kits (LAPUDA, China). Serum Indole-3-carboxaldehyde level was determined using commercially available ELISA kits (Boshen, China). All procedures were performed according to the manufacturer’s instructions. All samples (n=6) were analyzed in duplicate.

### 5.4 16S rDNA gene sequencing

The OMEGA Soil DNA Kit (D5625-01) from Omega Bio-Tek, located in Norcross, GA, USA, was utilized for the extraction of genomic DNA from a range of samples, followed by assessments of DNA purity and concentration. Amplicons encompassing the V3-V4 hypervariable regions were specifically synthesized through PCR, employing barcoded primers and high-fidelity DNA polymerase. The PCR products were then subjected to 2% agarose gel electrophoresis for visualization; targeted bands were excised and purified using the Quant-iT PicoGreen dsDNA Assay Kit. Initial measurements of these fragments from gel electrophoresis were followed by precise quantification using a fluorescence quantification system with a Microplate reader (BioTek, FLx800), and the samples were prepared for sequencing according to specific concentration ratios. Library construction was performed using the Illumina TruSeq Nano DNA LT Library Prep Kit and evaluated for quality with the Agilent Bioanalyzer 2100 and Promega QuantiFluor systems before sequencing.

Sequencing outputs were provided in FASTQ format. Adapter sequences from paired-end reads were identified and excised using Cutadapt software. Following adapter removal, the reads underwent quality control, denoising, merging, and chimera elimination using DADA2 within the default settings of QIIME2. The process culminated in the generation of a dataset comprising representative reads and a table of Amplicon Sequence Variant (ASV) abundances. We used the Shannon curve to evaluate sequencing depth. The Shannon curve is constructed based on the microbial diversity index at different sequencing depths for each sample. When the curve flattens, it indicates that the sequencing depth is sufficiently large to reflect the majority of microbial information present in the samples.

### 5.5 Analysis of serum metabolic profiles

Initially, 100 μL of each sample was transferred into an EP tube (n=6). To this, 400 μL of extraction solution (methanol with an isotopically-labeled internal standard mixture) was added. The samples were then vortexed for 30 seconds, sonicated for 10 minutes in an ice-water bath, and subsequently incubated at -40°C for one hour to precipitate proteins. Following this, the samples were centrifuged at 12,000 rpm (RCF=13800 ×g, R= 8.6 cm) for 15 minutes at 4°C. The supernatant was then decanted into a new glass vial for subsequent analysis. A quality control (QC) sample was prepared by pooling equal volumes of the supernatants from all samples.

The analyses were conducted using a Vanquish UHPLC system (Thermo Fisher Scientific) equipped with a UPLC HSS T3 column (2.1 mm × 100 mm, 1.8 μm) coupled to an Orbitrap Exploris 120 mass spectrometer (Thermo). The mobile phase comprised 5 mmol/L ammonium acetate and 5 mmol/L acetic acid in water (solvent A) and acetonitrile (solvent B). The autosampler was maintained at 4°C, and the injection volume was set at 2 μL.

The Orbitrap Exploris 120 mass spectrometer was employed for its capacity to perform MS/MS spectra acquisition in information-dependent acquisition (IDA) mode, managed by Xcalibur software (Thermo). This mode allows for continuous evaluation of the full scan MS spectrum. ESI source conditions were optimized with a sheath gas flow rate of 50 Arb, an auxiliary gas flow rate of 15 Arb, and a capillary temperature of 320°C. The resolutions for full MS and MS/MS were set at 60,000 and 15,000, respectively, with collision energies of 10/30/60 in NCE mode. Spray voltages were set at 3.8 kV for positive and -3.4 kV for negative ion modes.

The acquired raw data were converted to the mzXML format using ProteoWizard and processed with a custom-built R program, based on XCMS, for peak detection, extraction, alignment, and integration. An in-house MS2 database (BiotreeDB) was utilized for metabolite annotation, with a threshold set at 0.3 for inclusion.

### 5.6 Chemicals and reagents

Indole-3-carboxaldehyde (I3A, I106884, molecular formula: C9H7NO, relative molecular mass: 145.16, purity: >97%), Dehydroepiandrosterone (DHEA, D106380, molecular formula: C19H28O2, relative molecular mass: 288.43, purity: >99%) was purchased from Aladdin (Shanghai, China).

### 5.7 Cell culture and treatments

The KGN cell was maintained in Dulbecco’s Modified Eagle Medium/Nutrient Mixture F-12 (DMEM/F12) (KGL1201-500, Keygen Biotech, China), supplemented with 10% charcoal-stripped fetal bovine serum (Gibco) and 1% antibiotics (a combination of penicillin, streptomycin, and neomycin; Gibco). These cells were incubated at 37°C in a 5% CO2 atmosphere (Thermo Fisher Scientific, Waltham, MA, USA) and subcultured daily. For the I3A experiments, cells were collected following 24 hours of treatment. Additionally, cells were exposed to 20 μM DHEA. Both I3A and DHEA were dissolved in DMSO; thus, a comparable volume of DMSO was used for the control group.

### 5.8 CCK-8 assay

A CCK-8 assay was conducted using a commercial kit (Cat. No. C0005; TargetmoI) according to the instructions provided by the manufacturer. Cells were plated in 96-well plates at a density of 5,000 cells per well. At 24 and 48 hours post-seeding, 10μl of the CCK-8 solution was added to each well. The plates were then incubated for an hour, and absorbance at 450 nm was measured using a microplate reader. The assay was carried out in triplicate and repeated three times.

### 5.9 Western blot

The primary antibodies employed included Anti-Ampk-α (Cell Signaling, #2532), Anti-pAmpk-α Thr172 (Cell Signaling, #2535s), BAX (Proteintech, 50599-2-Ig), and BCL2 (Proteintech, 68103-1-Ig), with β-actin as a loading control (Proteintech, 66009-1-Ig, Wuhan, China). Secondary antibodies were sourced from Proteintech (SA00001-1, SA00001-2, Wuhan, China).

### 5.10 Quantitative real-time RT-PCR

Total RNA was isolated using the RNAeasy™ kit (R0027; Beyotime Biotechnology), adhering to the protocol provided by the manufacturer. Subsequently, the isolated RNA was reverse transcribed to complementary DNA (cDNA) utilizing the SuperMix (R323-01; Vazyme). Following the synthesis of cDNA, a quantitative polymerase chain reaction (qPCR) was performed using the Quantstudio 5 system with SYBR Green PCR Master Mix (Q311-02; Vazyme). For normalization purposes, β-actin was used as the internal control gene. Details of the primer sequences used can be found in **Supplementary Table S1**.

### 5.11 Statistical analysis

Data analysis was performed using prism. Results from a minimum of three independent experiments are presented as mean ± standard deviation. The Student’s t-test was applied to evaluate the differences in gene expression across groups, considering P < 0.05 as statistically significant.

## Data Availability

The original contributions presented in the study are included in the article/**Supplementary Material**. Further inquiries can be directed to the corresponding author.

## References

[1] Teede HJ Tay CT Laven JJE Dokras A Moran LJ Piltonen TT. Recommendations from the 2023 international evidence-based guideline for the assessment and management of polycystic ovary syndrome. J Clin Endocrinol Metab. (2023) 108:2447–69. doi: 10.1210/clinem/dgad463 PMC10505534 37580314

[2] Ye HY Song YL Ye WT Xiong CX Li JM Miao JH. Serum granulosa cell-derived TNF-α promotes inflammation and apoptosis of renal tubular cells and PCOS-related kidney injury through NF-κB signaling. Acta Pharmacol Sin. (2023) 44(12):2432–44. doi: 10.1038/s41401-023-01128-0 PMC10692080 37507430

[3] Qi X Yun C Sun L Xia J Wu Q Wang Y. Gut microbiota-bile acid-interleukin-22 axis orchestrates polycystic ovary syndrome. Nat Med. (2019) 25:1225–33. doi: 10.1038/s41591-019-0509-0 PMC7376369 31332392

[4] Kim D Yan J Bak J Park J Lee H Kim H. Sargassum thunbergii extract attenuates high-fat diet-induced obesity in mice by modulating AMPK activation and the gut microbiota. Foods (Basel Switzerland). (2022) 11:2529. doi: 10.3390/foods11162529 36010531 PMC9407432

[5] Lee HS Heo CU Song Y-H Lee K Choi C-I. Naringin promotes fat browning mediated by UCP1 activation via the AMPK signaling pathway in 3T3-L1 adipocytes. Arch Pharmacal Res. (2023) 46:192–205. doi: 10.1007/s12272-023-01432-7 36840853

[6] Zhang S-Y Lam TKT. Metabolic regulation by the intestinal metformin-AMPK axis. Nat Commun. (2022) 13:2851. doi: 10.1038/s41467-022-30477-3 35606343 PMC9126964

[7] Zhang E Jin L Wang Y Tu J Zheng R Ding L. Intestinal AMPK modulation of microbiota mediates crosstalk with brown fat to control thermogenesis. Nat Commun. (2022) 13:1135. doi: 10.1038/s41467-022-28743-5 35241650 PMC8894485

[8] Ma T Tian X Zhang B Li M Wang Y Yang C. Low-dose metformin targets the lysosomal AMPK pathway through PEN2. Nature. (2022) 603:159–65. doi: 10.1038/s41586-022-04431-8 PMC8891018 35197629

[9] Roland AV Moenter SM. Prenatal androgenization of female mice programs an increase in firing activity of gonadotropin-releasing hormone (GnRH) neurons that is reversed by metformin treatment in adulthood. Endocrinology. (2011) 152:618–28. doi: 10.1210/en.2010-0823 PMC3037157 21159854

[10] Hu R Long S Luo M Tang B Tan T Dong W. Hyperglycemia inhibits hepatic SHBG synthesis through the NGBR-AMPK-HNF4 pathway in rats with polycystic ovary syndrome induced by letrozole in combination with a high-fat diet. Mol Nutr Food Res. (2024) 68(14):e2300915. doi: 10.1002/mnfr.202300915 38862276

[11] Chakraborty S Anand S Coe S Reh B Bhandari RK. The PCOS-NAFLD multidisease phenotype occurred in medaka fish four generations after the removal of bisphenol a exposure. Environ Sci Technol. (2023) 57:12602–19. doi: 10.1021/acs.est.3c01922 PMC10469501 37581432

[12] Hu R Huang Y Liu Z Dong H Ma W Song K. Characteristics of polycystic ovary syndrome rat models induced by letrozole, testosterone propionate and high-fat diets. Reprod BioMed. (2024) 50:104296. doi: 10.1016/j.rbmo.2024.104296 39626468

[13] Jang KK Heaney T London M Ding Y Putzel G Yeung F. Antimicrobial overproduction sustains intestinal inflammation by inhibiting enterococcus colonization. Cell Host Microbe. (2023) 31:1450–1468.e8. doi: 10.1016/j.chom.2023.08.002 37652008 PMC10502928

[14] Delgado I Cussotto S Anesi A Dexpert S Aubert A Aouizerate B. Association between the indole pathway of tryptophan metabolism and subclinical depressive symptoms in obesity: a preliminary study. Int J Obes. (2022) 46:885–8. doi: 10.1038/s41366-021-01049-0 35001078

[15] Lu Y Yang W Qi Z Gao R Tong J Gao T. Gut microbe-derived metabolite indole-3-carboxaldehyde alleviates atherosclerosis. Signal Transduction Targeted Ther. (2023) 8:378. doi: 10.1038/s41392-023-01613-2 PMC10547776 37789009

[16] Shan S Qiao Q Yin R Zhang L Shi J Zhao W. Identification of a novel strain lactobacillus reuteri and anti-obesity effect through metabolite indole-3-carboxaldehyde in diet-induced obese mice. J Agric Food Chem. (2023) 71(1). doi: 10.1021/acs.jafc.2c05764 36786753

[17] Paeslack N Mimmler M Becker S Gao Z Khuu MP Mann A. Microbiota-derived tryptophan metabolites in vascular inflammation and cardiovascular disease. Amino Acids. (2022) 54:1339–56. doi: 10.1007/s00726-022-03161-5 PMC9641817 35451695

[18] Xie L-W Cai S Lu H-Y Tang F-L Zhu R-Q Tian Y. Microbiota-derived I3A protects the intestine against radiation injury by activating AhR/IL-10/wnt signaling and enhancing the abundance of probiotics. Gut Microbes. (2024) 16:2347722. doi: 10.1080/19490976.2024.2347722 38706205 PMC11086037

[19] Zhang T Xu D Trefts E Lv M Inuzuka H Song G. Metabolic orchestration of cell death by AMPK-mediated phosphorylation of RIPK1. Sci (New York NY). (2023) 380:1372–80. doi: 10.1126/science.abn1725 PMC10617018 37384704

[20] Mićić B Teofilović A Djordjevic A Veličković N Macut D Vojnović Milutinović D. AMPK activation is important for the preservation of insulin sensitivity in visceral, but not in subcutaneous adipose tissue of postnatally overfed rat model of polycystic ovary syndrome. Int J Mol Sci. (2022) 23(16):8942. doi: 10.3390/ijms23168942 36012206 PMC9408918

[21] Zuo M Liao G Zhang W Xu D Lu J Tang M. Effects of exogenous adiponectin supplementation in early pregnant PCOS mice on the metabolic syndrome of adult female offspring. J Ovarian Res. (2021) 14:15. doi: 10.1186/s13048-020-00755-z 33455575 PMC7812650

[22] Duca FA Côté CD Rasmussen BA Zadeh-Tahmasebi M Rutter GA Filippi BM. Metformin activates a duodenal Ampk-dependent pathway to lower hepatic glucose production in rats. Nat Med. (2015) 21:506–11. doi: 10.1038/nm.3787 PMC6104807 25849133

[23] Tobar N Rocha GZ Santos A Guadagnini D Assalin HB Camargo JA. Metformin acts in the gut and induces gut-liver crosstalk. Proc Natl Acad Sci USA. (2023) 120:e2211933120. doi: 10.1073/pnas.2211933120 36656866 PMC9942892

[24] Yang Y-L Zhou W-W Wu S Tang W-L Wang Z-W Zhou Z-Y. Intestinal flora is a key factor in insulin resistance and contributes to the development of polycystic ovary syndrome. Endocrinology. (2021) 162:bqab118. doi: 10.1210/endocr/bqab118 34145455 PMC8375444

[25] Gao N Yang Y Liu S Fang C Dou X Zhang L. Gut-derived metabolites from dietary tryptophan supplementation quench intestinal inflammation through the AMPK-SIRT1-autophagy pathway. J Agric Food Chem. (2022) 70:16080–95. doi: 10.1021/acs.jafc.2c05381 36521060

[26] Davidson DJ Currie AJ Reid GSD Bowdish DME MacDonald KL Ma RC. The cationic antimicrobial peptide LL-37 modulates dendritic cell differentiation and dendritic cell-induced T cell polarization. J Immunol (Baltimore Md: 1950). (2004) 172:1146–56. doi: 10.4049/jimmunol.172.2.1146 14707090

[27] Tremblay S Côté NML Grenier G Duclos-Lasnier G Fortier L-C Ilangumaran S. Ileal antimicrobial peptide expression is dysregulated in old age. Immun Ageing: I A. (2017) 14:19. doi: 10.1186/s12979-017-0101-8 28855949 PMC5575895

[28] Aldhous MC Noble CL Satsangi J. Dysregulation of human beta-defensin-2 protein in inflammatory bowel disease. PloS One. (2009) 4(7):e6285. doi: 10.1371/journal.pone.0006285 19617917 PMC2708916

[29] Li Y Peng Y Yang Y Shi T Liu R Luan Y. Baicalein improves the symptoms of polycystic ovary syndrome by mitigating oxidative stress and ferroptosis in the ovary and gravid placenta. Phytomedicine. (2024) 128:155423. doi: 10.1016/j.phymed.2024.155423 38518646

