# Supplementary material for: Metformin-mediated intestinal AMPK activation ameliorates PCOS through gut microbiota modulation and metabolic pathways
Source: Front Endocrinol (Lausanne). 2025 Feb 18;16:1526109. doi: 10.3389/fendo.2025.1526109 (PMC11876036; doi:10.3389/fendo.2025.1526109)
Supplement: Supplementary file 1 [file Table1.docx]

Supplementary Materials for

Intestinal AMPK regulates microbiota and serum metabolites in the treatment of PCOS

Xu Yating, Si yu, Ning Li, Li Xiu, Wang Ruyue, Zhao Hongting, Ren Qingling

Correspondence to:

**This PDF file includes:**

Supplemental Table S1 Primer sequence

Supplemental Table S1 Primer sequence

| **Gene** | **Primer(5'to3')** |
| --- | --- |
| *h-bactin-F* | GCGGGAAATCGTGCGTGAC |
| *h-bactin-R* | CAGGAAGGAAGGCTGGAAGAGTG |
| *r-bactin-F* | CTAAGGCCAACCGTGAAAAG |
| *r-bactin-R* | ACCAGAGGCATACAGGGACA |
| *bax-F* | TGCTAGCAAACTGGTGCTCA |
| *bax-R* | TGTCCAGCCCATGATGGTTC |
| *r-bcl2-F* | CTGAGTACCTGAACCGGCAT |
| *r-bcl2-R* | AGGTATGCACCCAGAGTGATG |
| *h-bcl2-F* | GACTTCGCCGAGATGTCCAG |
| *h-bcl2-R* | GAACTCAAAGAAGGCCACAATC |
| *r-defa6-F* | ATTTTGAGATAGGAAGGCCAGT |
| *r-defa6-R* | TAAATGGACCTTGAGCACAGAA |
| *r-reg3a-F* | GTCTGTCCTTCAACAATGTGTC |
| *r-reg3a-R* | GCAGTAAGAACGATAAGCCTTG |
| *r-reg3g-F* | TTCTTCTGTCTCTGGAAGTCAC |
| *r-reg3g-R* | GCAGACATATGGCAATTCTGAG |
| *r-Lyz2-F* | AATGGGATGTCTGGCTACTATG |
| *r-Lyz2-R* | GTCATTACACCAGTATCGGCTA |
