# Supplementary figures and images for: Metformin-mediated intestinal AMPK activation ameliorates PCOS through gut microbiota modulation and metabolic pathways
Source: Front Endocrinol (Lausanne). 2025 Feb 18;16:1526109. doi: 10.3389/fendo.2025.1526109 (PMC11876036; doi:10.3389/fendo.2025.1526109)

Ileum AMPK(62kD) p-AMPK(62kD)

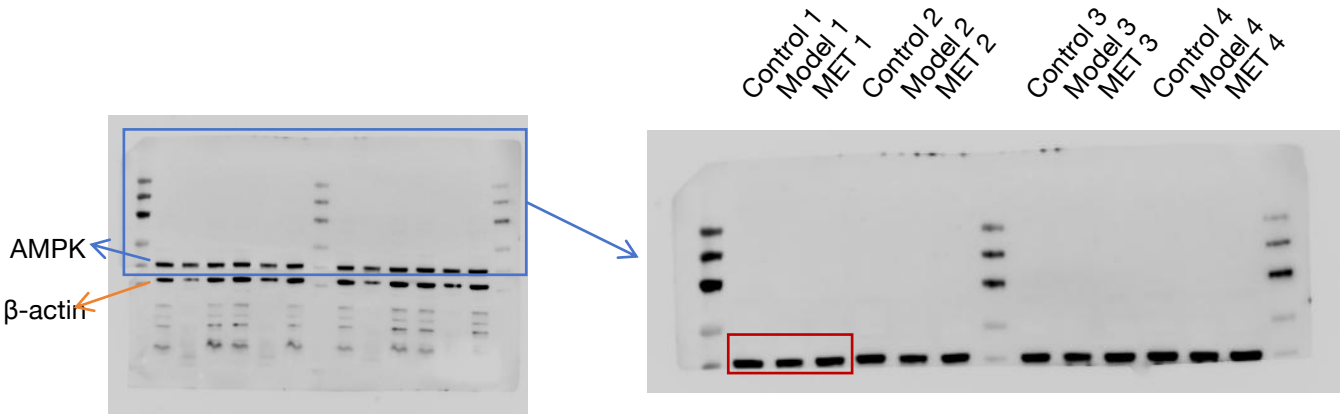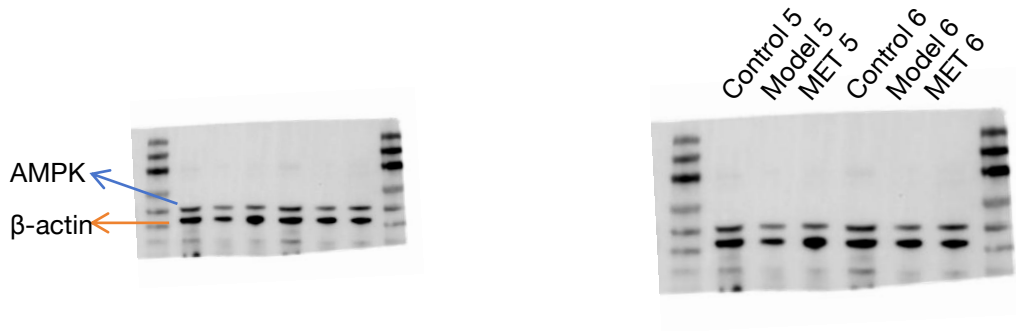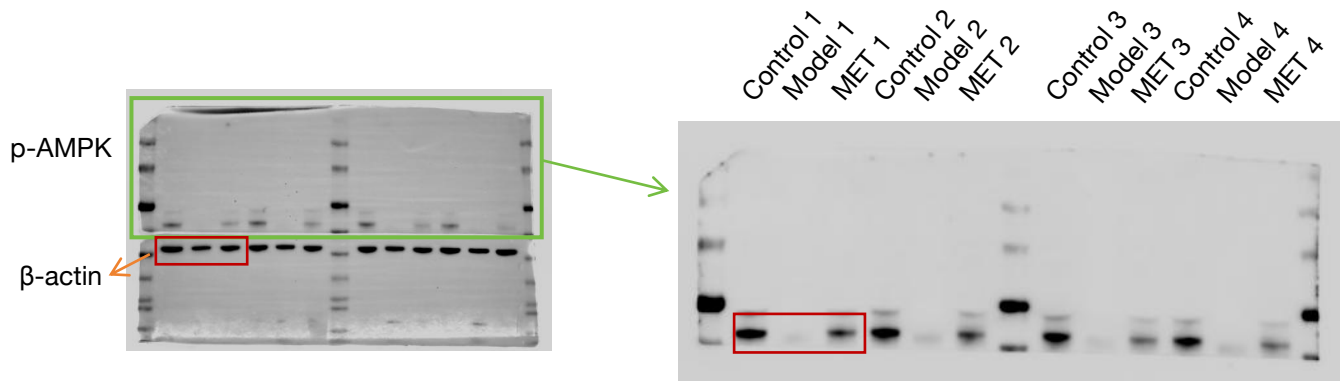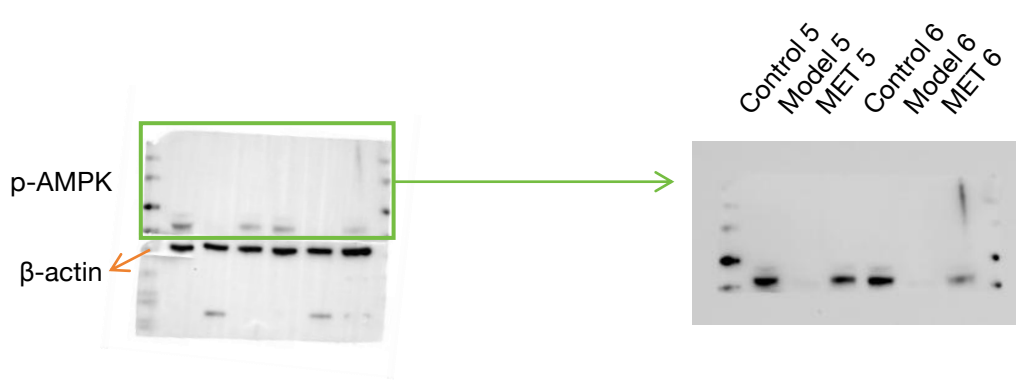

Ovary Bax(21kD) Bcl2(26kD)

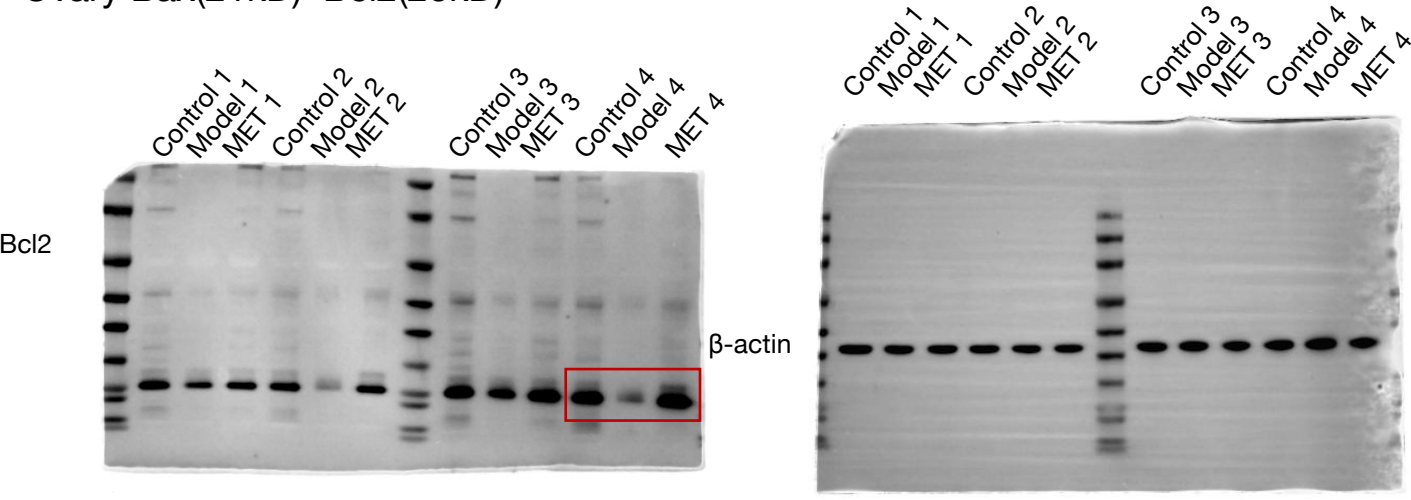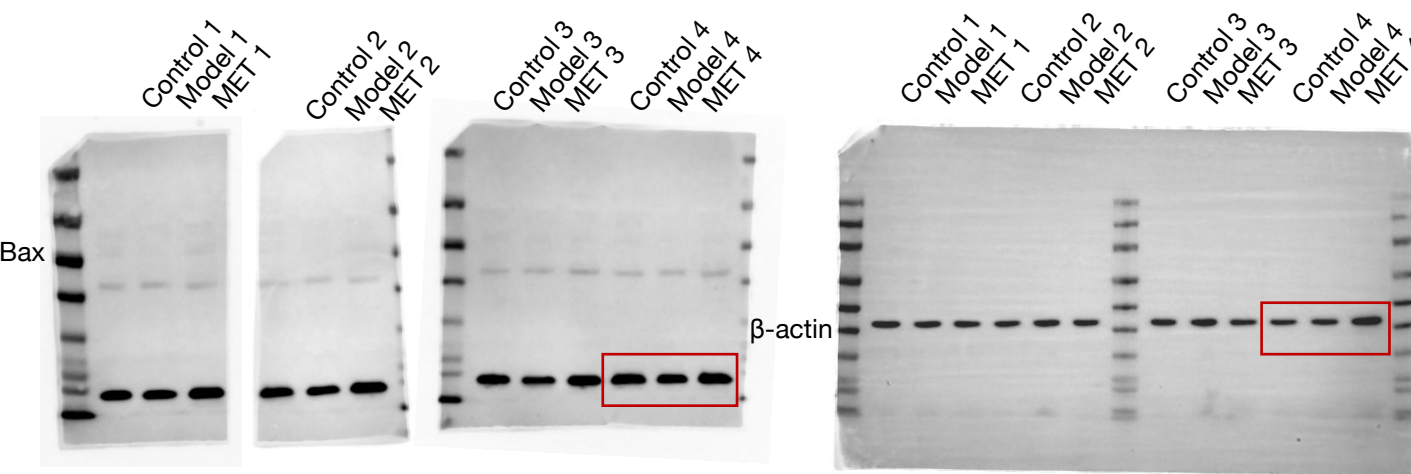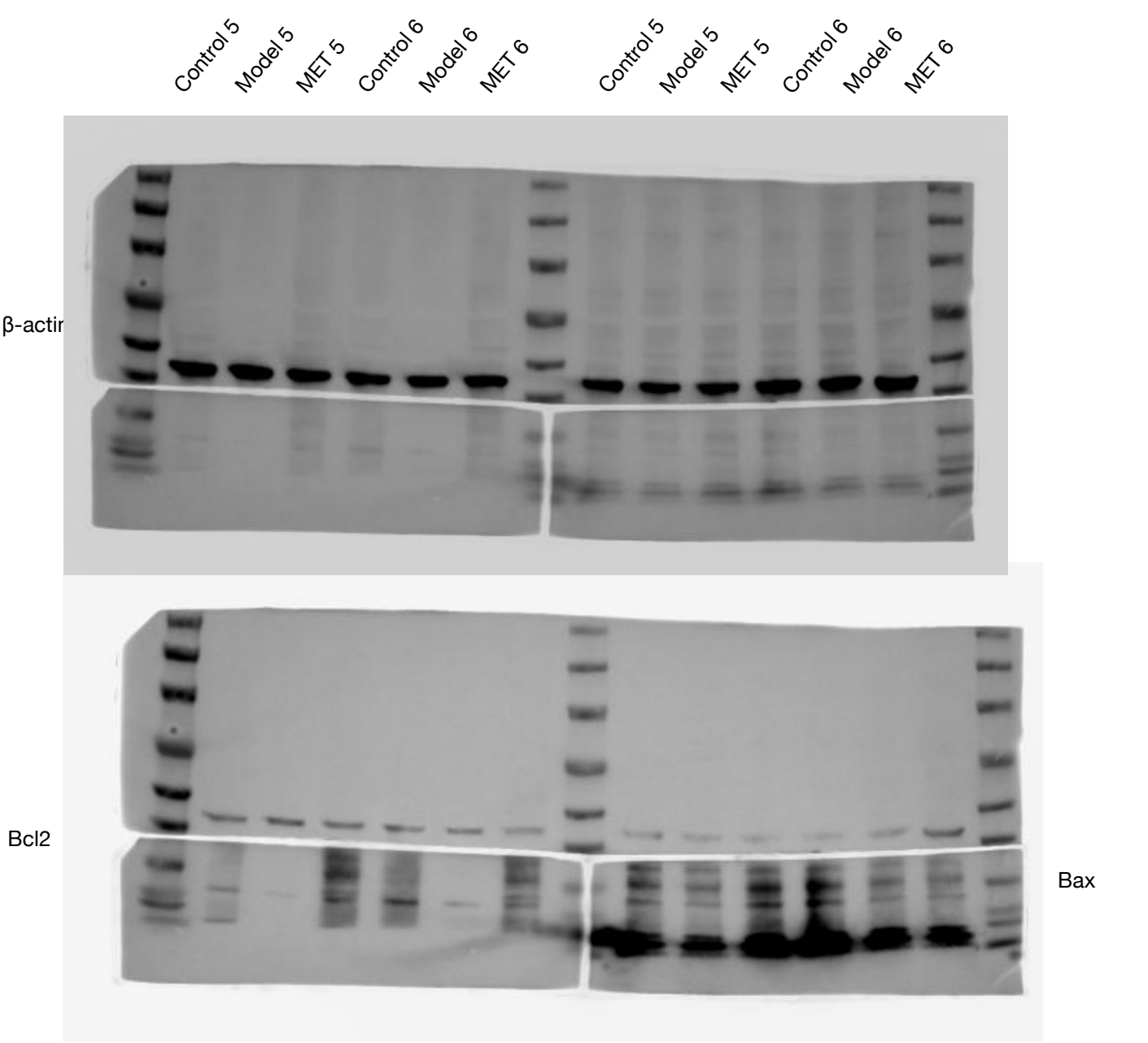

KGN

Bax(21kD) Bcl2(26kD)

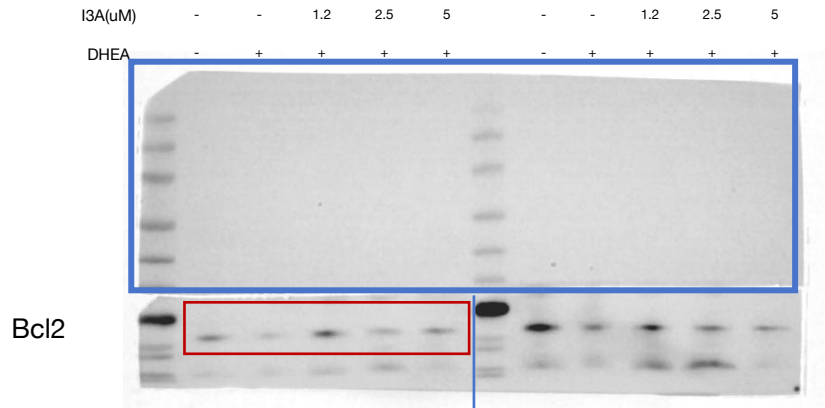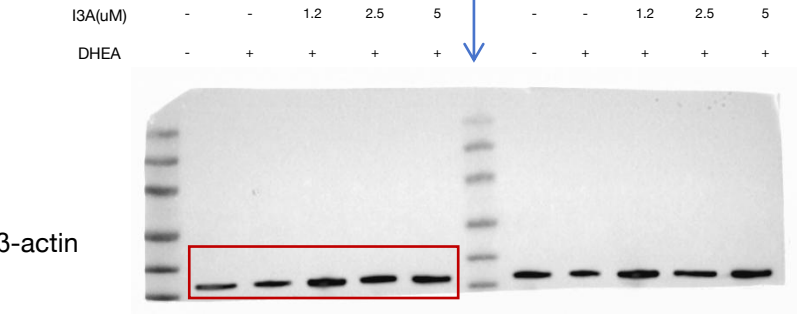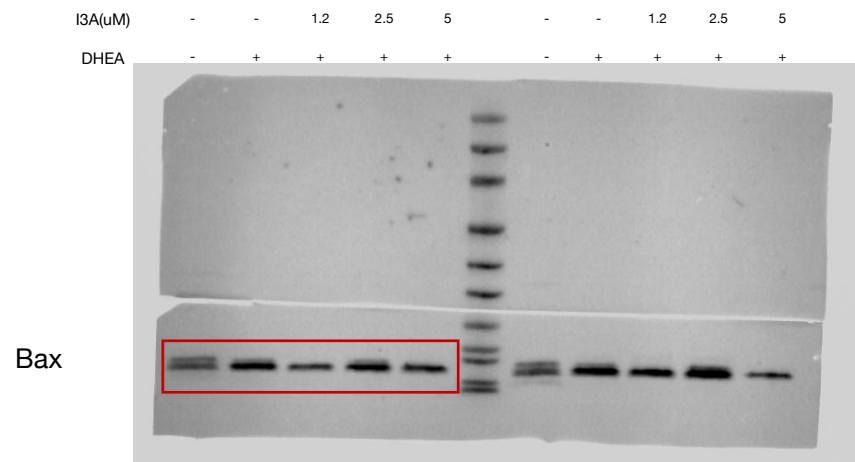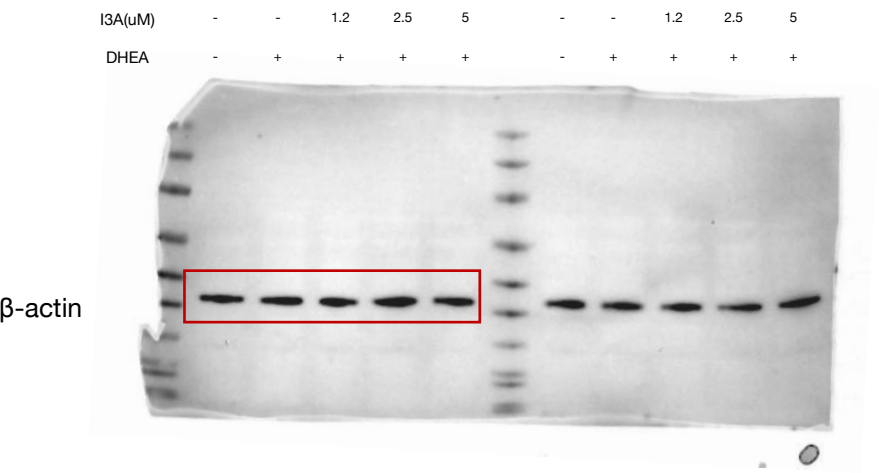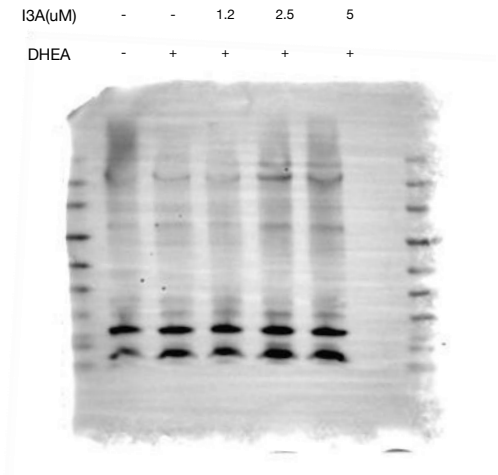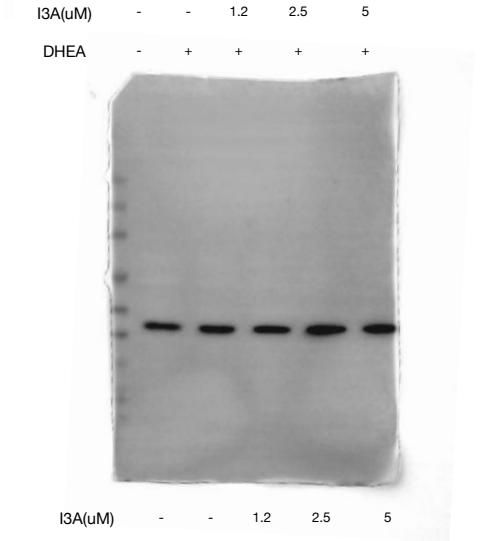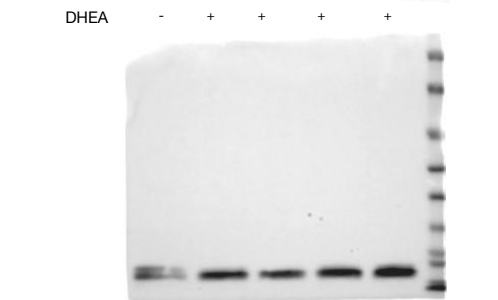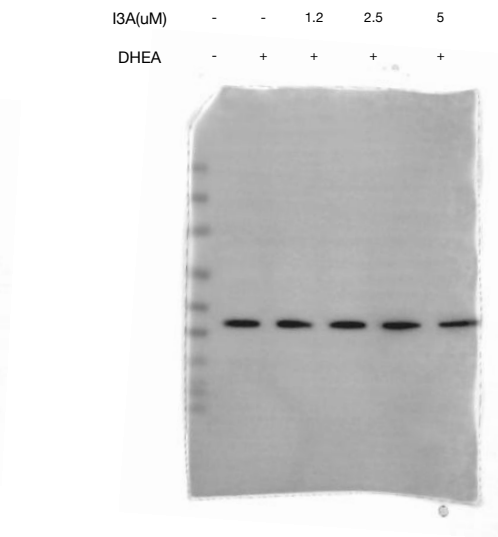

Supplement: Supplementary file 2 [file DataSheet1.pdf]
